# Current State of Global African Swine Fever Vaccine Development under the Prevalence and Transmission of ASF in China

**DOI:** 10.3390/vaccines8030531

**Published:** 2020-09-15

**Authors:** Keke Wu, Jiameng Liu, Lianxiang Wang, Shuangqi Fan, Zhaoyao Li, Yuwan Li, Lin Yi, Hongxing Ding, Mingqiu Zhao, Jinding Chen

**Affiliations:** 1 College of Veterinary Medicine, South China Agricultural University, Guangzhou 510642, China; 13660662837@163.com (K.W.); gemini1996@stu.scau.edu.cn (J.L.); hnndfsq@126.com (S.F.); lzhaoyao123@163.com (Z.L.); lyw13253374768@163.com (Y.L.); yilin@scau.edu.cn (L.Y.); dinghx@scau.edu.cn (H.D.); zmingqiu@scau.edu.cn (M.Z.); 2 Guangdong Laboratory for Lingnan Modern Agriculture, Guangzhou 510642, China; 3 Hog Production Division, Guangdong Wen2019s Foodstuffs Group Co, Ltd., Xinxing 527439, China; animsci@126.com

**Keywords:** African swine fever, ASFV, China, epidemic situation, vaccines, progress

## Abstract

African swine fever (ASF) is a highly lethal contagious disease of swine caused by African swine fever virus (ASFV). At present, it is listed as a notifiable disease reported to the World Organization for Animal Health (OIE) and a class one animal disease ruled by Chinese government. ASF has brought significant economic losses to the pig industry since its outbreak in China in August 2018. In this review, we recapitulated the epidemic situation of ASF in China as of July 2020 and analyzed the influencing factors during its transmission. Since the situation facing the prevention, control, and eradication of ASF in China is not optimistic, safe and effective vaccines are urgently needed. In light of the continuous development of ASF vaccines in the world, the current scenarios and evolving trends of ASF vaccines are emphatically analyzed in the latter part of the review. The latest research outcomes showed that attempts on ASF gene-deleted vaccines and virus-vectored vaccines have proven to provide complete homologous protection with promising efficacy. Moreover, gaps and future research directions of ASF vaccine are also discussed.

## 1. Introduction

African swine fever (ASF) is a highly contagious animal infectious disease caused by African swine fever virus (ASFV) infecting both wild (*Sus scrofa*) and domestic swine of all breeds and ages. ASF with the acute form has a rapid onset and a short course of disease, which are characterized by high fever, loss of appetite, cyanosis, severe bleeding of internal organs and a high mortality rate of nearly 100% [1,2]. The less virulent strains can lead to mild clinical signs, subacute forms of disease, or even chronic infections.

ASFV is the sole member of the *Asfarvirus* genus within the *Asfarviridae* family, and is the unique Arbovirus whose genome is double-stranded DNA, ASFV genome shows significant variations in length from 170 to 194 kb, which mainly results from gain or loss of different members of multigene families (MGF) [3], and encodes 150–167 proteins including structural and host immunomodulatory proteins [4,5]. Morphologically, the ASFV virion is a symmetrical icosahedral particle with a diameter of about 200 nm and has a complex multi-enveloped [6]. ASFV replicates predominantly in mononuclear-phagocytic cells (monocytes and macrophages), and modulation of macrophage function by the virus is crucial for the pathogenic and immune evasion mechanisms [7].

**Figure 1:**
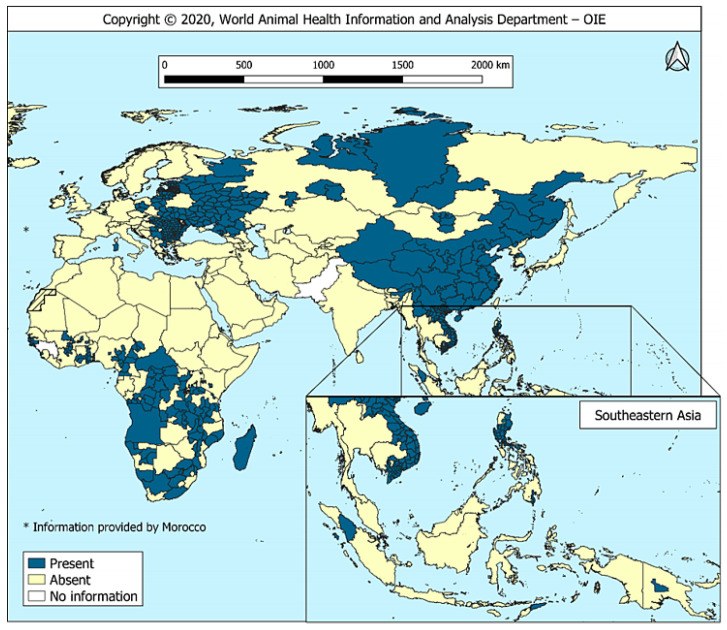
Global situation of ASF, Source: OIE, (2016–2020) [16].

The genome of ASFV is huge and complex, showing obvious genetic diversity. There are currently eight serotypes based on the viral hemagglutinin CD2-like protein (CD2v) and C-type lectin. According to the sequence of B646L, which encoding the major capsid protein p72, ASFV can be divided into 24 genotypes (I-XXIV) [8]. ASFV was first described in 1920s, and all the above genotypes can be detected in Africa. The genotype I ASFV emerged in Europe in 1950s and was eradicated in Europe except Sardinia by the mid-1990s. The genotype II ASFV was mainly prevalent in southeastern Africa. In 2007, it was introduced to Georgia, then continued to spread across the Caucasus region and into Russian Federation and Eastern Europe [9,10]. In early March 2017, the genotype II strain spread eastward across a long distance to Irkutsk in Russia’s Far East region and near the border of China [1]. Since August 2018, a highly virulent genotype II ASFV has been spread to China [11,12,13] and successively to Mongolia, Vietnam, Cambodia, Laos, North Korea, Laos, Philippines, Myanmar, South Korea, Indonesia, and other Asia-Pacific countries [14], as is shown in Figure 1. In addition, the first ASF occurrence have been reported in Serbia, Greece, and other European countries [15]. 

Recently, the World Organization for Animal Health (OIE) issued a report on the “Global Situation of ASF” which indicated that there has been a marked pattern of increasing ASF outbreaks worldwide since 2016. As of 18 June 2020, 30% (60/201) of the reporting countries/regions had reported the presence of the disease, Since 2020, 8330 ASF outbreaks have occurred in 25 countries and regions worldwide, including 1604 swine and 6726 wild boars, showing a serious deterioration in the incidence of the disease, especially in Europe and Asia. Europe accounted for 67% of the outbreaks reported through immediate notifications and follow-up reports, notably, Asia (6,733,791 animals lost, which is 82% of the total global reported losses) accounted for the largest proportion of reported pig losses. OIE encourages interregional cooperation to combat the spread of this cross-border transmitted disease [16].

At present, effective control of ASF has not yet been achieved in Asia and Europe, which poses a serious threat to the global pig industry. No available vaccines against ASF leaves the control of the disease to early detection by efficient diagnosis, the culling of infected and exposed animals, and strict sanitary measures. Lack of in-depth understanding of the prevalence, transmission mechanism and pathogenicity of ASF has brought great difficulties to the prevention and treatment of ASF in China. The purpose of this review is to summarize the current epidemic situation and factors of ASF in China. The second half of this review covered the recent progress and emerging approaches to ASF vaccine design. We look forward to delivering helpful information for the next generation of ASF vaccines to leap over hurdles.

## 2. Epidemic Situation of ASF in China

**Figure 2:**
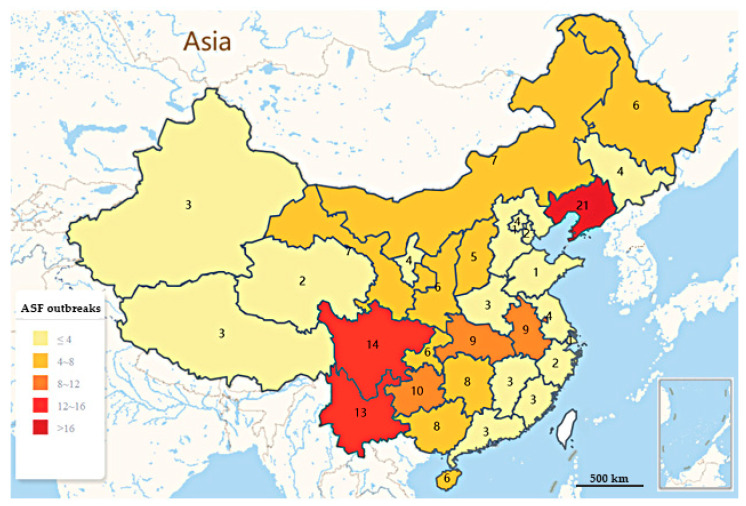
The distribution of ASF outbreaks in China (August 2018–July 2020). Outbreak analysis of geographical distribution showed that as of 24 July 2020, 178 outbreaks were reported in 31 provinces and geographical regions of China (Figure 2), and the outbreak rates of ASF in each province are shown in Table 1. Comparison of the provinces showed that the spread tendency of the outbreaks is mainly concentrated on the northeast and southwest regions, and the trend is increasing southward [18].

ASF has brought a grievous blow to China, which is the world’s largest pork producer and consumer, with pork production accounting for about 53% of the world’s supply. Further, pork is the main source of high-quality protein for Chinese residents, with the consumption accounting for 62% of total meat consumption [1]. Until 2017, the pig industry in China has been modernizing and expanding, and it is estimated that there will be about 700 million pigs annually. Nevertheless, the first ASF outbreaks in China was declared in Shenbei New Area, Shenyang, Liaoning in August 2018 [11,12,13]. Zhou et al. [13] found that ASFV-SY18, the first outbreak strain diagnosed in China, belonged to the genotype II group and shared 100% nucleotide identity with the strains isolated in Georgia, Russia, and Estonia based on the p72 gene fragment, which indicated the close relationship among these viruses. Within a few months after the first ASF outbreak, it quickly swept through most provinces in China. By 24 July 2020, Ministry of Agriculture and Rural Affairs of the People’s Republic of China (MARA) has reported 178 ASF outbreaks (including four outbreaks in wild boar) in 31 provinces, municipalities and autonomous regions (the ASF outbreaks distribution map in China is shown in Figure 2, The outbreaks were mainly distributed in major economic zones with frequent trade of pig industry, with a trend of increasing southward on the whole. As is shown in Table 1, the southwest region was the most severely affected with 25.84% (46/178) of outbreaks, followed by the northeast region with 16.85% (30/178) of outbreaks. Among the region Heilongjiang accounted for the largest cumulative number of reported susceptible and dead pigs [17,18]. At least 1.2 million pigs have been culled, causing tens of billions of direct economic losses. According to statistics from the National Bureau of statistics of China, in 2019, the number of live pigs and pork production in China were both decreased year on year. Compared with the same period last year, with 544 million live pigs, the number of live pigs dropped 21.6%, and pork production was 42.55 million tons, lower 21.3%. In the first quarter of 2020, affected by the persistent impact of ASF, the number of live pigs in the country was 131.29 million, which was 57.14 million less than the same period last year, dropped 30.3%; and the pork production was 10.38 million tons, which was 4260 million tons less than the same period last year, dropped 29.1% [19,20,21]. In 2019, China’s pork export volume was 210,000 tons, a year-on-year decrease of 36.17%, which has tremendous consequences for international trade. In the face of strong and sustained demand, the available pork supply has subsequently declined, leading to a substantial increase in the price of pork sold on the domestic market, from 12.2 yuan per kg (February 2019) to 36.1 yuan per kg in February 2020 [22]. The outbreak of ASF caused a considerable loss of pig capacity in China, and the pig industry has suffered a devastating blow. It is expected that it will be difficult to recover to its previous level within 3–5 years [23]. Due to the concealment and complexity of ASFV transmission, the epidemic situation in China is still not optimistic. 

## 3. Factors of ASF Prevalence in China

ASF has been listed as a foreign animal disease with focused surveillance all along in China. After the first report of the ASF epidemic on 3 August 2018, the MARA immediately initiated an emergency response and took various preventive and control measures in time, but still failed to curb its spread. The following analyzed the influencing factors of the ASF epidemic in China.

### 3.1. Strong Environmental Resistance of ASFV

The ASFV genotype II strain prevalent in China has strong pathogenicity and infectivity, and is highly contagious and stable in the environment [12]. The virus can be long-term survival and be infectious in the environment with pH 4–10, taking 70 min at 56 °C, 20 min at 60 °C to inactivate it. It can survive for more than one year in blood stored at 4 °C, persist for 11 days in swine feces, survive for up to one year in dead wild boar carcasses, and for a long time (months, or even years) in processed (cured, smoked, or uncooked) or frozen pork, and spread with the circulation of pork products [24].

### 3.2. Role of Viral Circulation in the Occurrence of ASF

ASFV has complex and diverse transmission mode, it can circulate among domestic pigs-wild boars, wild boars-soft ticks-pigs, and can be propagated in pigs through direct and indirect contact or short distance aerosol [7,25]. 

ASFV can be transmitted horizontally in infected pigs. The virus content in the blood of highly acute attack pigs is very high, which provides favorable conditions for the direct or indirect transmission of the virus. The virus also exists in urine, nasal secretions, saliva and other excreta and spreads through short distance aerosol. In addition, direct contact with contaminated surfaces, feed feces or water can indirectly lead to pig infection. All of these pathways contribute to the persistence of ASFV in pigs [26]. Although the mortality rate of genotype II ASFV is extremely high, the surviving infected pigs will become clinically healthy virus carriers, which may become a source of new acute infections [25,27].

Wild boars have a wide scope of activities, ASFV can survive in infected wild boar carcasses and environment for months, and mechanical transmission of pathogens will occur once the carcasses are eaten by other carnivores or birds. Different from warthog and giant forest pigs in Africa, Eurasian wild boar (*Sus scrofa*) lack resistance to ASFV and have a high mortality rate once infected. Since 2014, 95% of reported ASF outbreaks in Europe have been wild boar infections, and ASF persistence has been maintained through transmission between wild boars in the absence of domestic boars [28]. Four ASF outbreaks in wild boar have been reported so far in China. On 16 November 2018, the first outbreak in wild boar was confirmed in Hunjiang District, Baishan City of Jilin Province, and though there was no direct connection with domestic pig infection, the source of infection remains unknown [29]. In China, wild boar population is quite large, including both natural and farmed wild boars, whose density is estimated to be 2–5/km^2^, and widely distributed in Fujian, Guangdong, Northeast China, Yunnan-Guizhou region, etc. It is estimated that the number of wild boars in the whole country has reached millions now [30]. Despite the reported outbreaks in China mainly in swine, the potential risk of ASFV transmission from wild boars to swine is high based on the huge population and wide distributed of wild boar in China. Wild boar, as a natural host, once intervened in the infection cycle chain, it is likely to form a natural epidemic focus, which would greatly increase the control difficulty and is not conducive to the eradication of ASF [31].

ASFV can persist in soft ticks for a long time and be infectious. Susceptible pigs are infected by ingesting soft ticks carrying ASFV or their bites [32]. Ticks widely distributed in South America can transmit ASFV, and Pereira et al. have confirmed the potential role of European soft ticks as hosts of ASFV and demonstrating the effectiveness of their unconventional transmission routes [33]. The ASF epidemic in China is characterized by endemic occurrence. Though there is no report on the ASF outbreaks caused by tick transmission in China yet. However, there are more than 100 species of ticks in China. Once the virus is carried by ticks, it will be more difficult to eradicate, and the risk of long-term existence of the virus will increase accordingly. The study on the role and mechanism of ticks in the transmission of ASF in China will be conducive to take effective measures to control the ASF epidemic in time.

### 3.3. Role of Human Activity in the Transmission of ASFV

Epidemiological studies of 68 outbreaks from August to November 2018 found that 19% of ASF outbreaks were caused by trans-regional transportation of live pigs and pork products, 46% by vehicles and movement of people, and 34% by swill feeding, revealing the important role human activities play in the transmission of ASFV [34].

Trans-regional transportation of live pig and pork products is an important consideration for the rapid spread of ASF in China. The pig trade occupies a large market share of breeding industry in China. Due to the uneven distribution of pig origin in China, line-haul of pigs cannot be avoided. In addition, the early diagnosis of ASF is difficult and its clinical symptoms are not obvious, easy to confuse with diseases such as Classical Swine Fever (CSF). Line-haul of infected pigs directly results in rapid spread of the virus. Legal trade of live pigs may spread ASF during high-risk periods (HRP). Gao Xiang et al. found that the southeast of China had the highest risk of ASFV transmission through legal pig trade, especially in colder months [35]. Therefore, in order to avoid the expansion of the epidemic scope, off-site transportation of live pigs and pork products is forbidden in many places. The limited transportation of live pigs immediately led to a rise of pig prices, and illegal pig transportation and frozen meat smuggling, which further resulted in the long-distance transmission of ASFV. In view of this, the MARA had strengthened the supervision and management of the transfer of pigs and their products, implemented measures such as registration and filing of pigs transport vehicles, inspection of transportation links and detection of slaughter links to crack down on illegal activities. Controlling the movement of pigs and transport of products seems to be effective, resulting in a decrease in the proportion of outbreaks from 35% to 15% [5,36].

According to previous statistics, domestic pig circulation is the main route of ASF transmission in China. Infection can occur when feeds and swill contaminated with ASFV from epidemic areas are ingested in healthy pigs [11]. Most of the swill comes from canteens or restaurants, and the water after cleaning pork may be contaminated by ASFV. Consequently, the MARA issued a ban on feeding food residues to pigs. At present, due to the prohibition of using kitchen waste to feed pigs, the proportion of outbreaks caused by feeding kitchen waste had dropped from 50% to 44% [17].

### 3.4. Analysis on Breeding Pattern of Diseased Pig Farms

In developing countries, most pig breeding remains traditional, small-scale, and self-sufficient. Small-scale family farming plays a major role in the introduction, dissemination and colonization of ASF due to its low biosecurity, backward feeding methods and techniques, insufficient understanding of animal health regulations, or even non-compliance with regulations [37]. At present, pig farming in China is in the transition stage from bulk culture to large-scale farming, with 26 million pig farm households. Although the proportion of large-scale farming is increasing year by year, less than 1% of pig farms have more than 500 sows annually, and the small-scale farming model will still exist for a long time. The first outbreak of ASF in China was detected in domestic pigs in Shenyang [12], contact tracing and field investigations found that illegal transportation and a lower biosecurity level played a significant part in the whole transmission process [13]. Small and medium-scale farms with low biosecurity levels in China have brought great challenges to ASF epidemic control, created conditions for the breeding and spread of ASF, increased great difficulties and burdens for epidemic prevention personnel to trace the epidemic situation. Fifty-four percent of the reported outbreaks in 2018 were from small- and medium-sized farms, further illustrating the lack of biosecurity control in individual scattered households [19]. The diversification of breeding modes, high feeding density and weak biosafety awareness of practitioners have aggravated the pressure on the prevention and control of ASF in China. Therefore, more attention should be paid to improving animal husbandry and raising biosecurity levels [22,38].

## 4. Chinese Model of ASF Control Strategy

There is no fixed model for the prevention and control of ASF, a Chinese model for the control of ASF is being established with absorbing and drawing on the experiences of foreign countries. At present, due to the decline of pig stocks, and benefited from the improvement of biosafety awareness and biosecurity level of pig farms in large scale, ASF epidemic has been monitored and detected by a large number of breeding enterprises, and it is slowing down in China [34]. However, ASFV still exists in some pig herds and pig farms in some regions, and the epidemic of ASF in China is a constant reality [19]. Overall, the situation of prevention and control is still grim.

Currently, there is no safe, effective and commercially available ASF vaccines, so there is no way to control susceptible animals. The prevention and control of ASF in China focuses on eliminating the contagium and polluter, and cutting off the transmission route, which will be a long-term work. Contagium and polluter mainly include dead and infected pigs, especially the infected pigs without clinical symptoms, which should be culled and harmlessly treated, and forbidden to enter the transportation, sale, and slaughtering links. It is necessary to strengthen the cleaning and disinfection of transport vehicles and the isolation of personnel; drying, high temperature granulation, acidification of polluted feed materials; drying and disinfection of polluted materials, disinfection and acidification of polluted water sources, cleaning and maturation of polluted food materials, and the measures mentioned above are intended to completely inactivate ASFV in the environment. In addition, appropriate measures should be taken to prevent the mechanical transmission of ASFV by fly/mosquito/bird. Finally, transport, slaughter, and feed enterprises, meat processing enterprises should do a good job in the biosecurity system of ASF and adopt some corresponding technologies to eliminate potential pollution sources [5,39].

New pig farms should meet the biosecurity requirements for the prevention and control of ASF, adhering to the general principles of “Scientific Design, Zonal Layout, Physical Isolation, Zonal Grading, Intelligent Management”. In order to prevent the spread of ASFV in pig farms and control the epidemic to a minimum extent and scope, we should strengthen the construction of the biosecurity system in pig farms and strictly implement the corresponding sanitary measures [40], which mainly includes the following six links:

(1) Introduction control. Introduced breeding pigs must be isolated for more than 30 days and tested clinically and laboratory to ensure that ASFV was negative.
(2) Transport vehicle control. Establishing a standardized cleaning and disinfection system and specifications for transport vehicles, and separate and specific trucks are recommended to transport the pig and material.
(3) Control of items entering pigsties. Strict disinfection treatment of items entering pig farms by fumigation, ozone and other methods.
(4) Personnel control. Personnel inside the pig farm should reduce their outgoing activities and isolation measures for admission personnel should be implement strictly.
(5) Feed control. Eliminate potential sources of contamination of feed.
(6) Environmental control. The surrounding environment of pig farms should be regularly monitored, detected and evaluated.

Moreover, the OIE also encourages members to implement enhanced national sanitary measures on waste disposal from aircrafts/vessels/passengers and enhanced in-farm biosecurity measures–including the protection of pigs from untreated swill feeding and the effective separation between domestic pigs and wild boar–and stresses the importance of OIE international standards for risk management of transboundary animal diseases (TADs) to reduce the risk of exporting disease to trading partners [16]. 

## 5. Current State of ASF Vaccine Development

Although ASFV was discovered as early as the beginning of the 20th century, vaccine research has lagged behind for more than 40 years, which may be related to its obvious genetic diversity, showed by the very large and complex genome of ASFV. Thus far, the exact mechanism of the host protective response has not been fully determined and protective antigens remain to be identified, which seriously hinders the development of vaccines. The lack of effective treatments or vaccines against ASF complicates the control of ASF in China. Consequently, we reviewed the research history and recent progress of ASF vaccines in order to provide reference for the research and development of ASF vaccine in China. Despite the obstacles, a few laboratories devoted to ASF vaccine research have made several pioneering discoveries in this field. For example, some candidate strains of gene-deleted vaccines can provide complete cross-protection, and are expected to be put on the market in the near future [41]. Furthermore, significant breakthroughs have also been made in the research of virus vector vaccines, which, for the first time, achieved complete protection of vaccinated pigs against virulent strains [42].

### 5.1. Inactivated Vaccines

Inactivated ASF vaccines were first attempted in 1960s, but studies have shown that inactivated ASFV as a vaccine is essentially an infeasible strategy [43]. In 2014, ASFV-specific antibodies were detected in piglets vaccinated with binary ethyleneimine (BEI)-inactivated ASFV Armenia08 strain in combination with the latest Polygen^TM^ or Emulsigen^®^ -D adjuvant, but the strain failed to provide protection and acute clinical symptoms were quickly observed [44]. Inactivated vaccines are antigenic but lacked the capability to stimulate the body to produce complete cellular immune responses, which may be part of the reasons why inactivated vaccines cannot provide immune protection [45].

### 5.2. Live Attenuated Vaccines (LAVs)

#### 5.2.1. Conventional LAVs

ASFV attenuated by continuous subculture can induce protective immune response against parental strains. In 1960s, the attenuated strain was widely used in Spain and Portugal, but it was abandoned due to the chronic infection symptoms of ASF, which extensively appeared in vaccinated pigs. Nevertheless, The ASFV Georgia strain, which were fully attenuated after 110 consecutive passages by Vero cells, cannot resist the attack of the parental ASFV Georgia virulence after immunizing pigs, and may not produce an effective immune response due to the change of its antigen during passage [46]. In the follow-up study, pigs inoculated with a naturally-attenuated strain NH/P68 of ASFV were resistant to the virulent strain ASFV L60, with the activity of NK cells increased, indicating that NK cells activity is positively correlated with the protective immunity of ASFV [47]. Moreover, vaccination with the naturally attenuated strain OURT88/3 could induce cross-protective immunity against attacks by non-homologous ASFV virulent strains, which is associated with the IFN-γ-secreting ability of lymphocytes in vaccinated pigs [48]. However, pigs vaccinated with naturally-attenuated ASF vaccines generally have many serious side effects, such as fever, abortion, and chronic or persistent infections [49]. In addition, there are also safety issues with virulence reversion. In the latest study in May 2020, an attempt was made to increase the safety of the naturally attenuated strain OURT88/3 by deleting I329L, a gene previously shown to inhibit the innate immune response of the host. Unexpectedly, the OURT88/3∆I329L strain significantly reduced protection against virulent stains OURT 88/1 attacks [50]. To date, the hinge to immune protection of naturally LAVs still needs to be further elucidated. For the current circumstances of ASF in China, the use of conventional LAVs should be cautious or forbidden. If there are no matching technologies and products of differentiating infected from vaccinated animals (DIVA), it is largely unfavorable for the eradication of ASFV in China.

#### 5.2.2. Recombinant LAVs/Gene-Deleted Vaccines

ASFV encodes a variety of proteins to inhibit and evade the host immune response by regulating host cell protein expression, interfering with the innate immune system and regulating cell cycle, which creating favorable conditions for self-proliferation and proliferation (Table 2). An appropriate balance between attenuation and immunogenicity may be achieved by exploring ASFV-encoded genes involved in replication, virulence, and immune evasion, which is conducive for the design of rationally gene-deleted vaccines. Additionally, technologies including reverse genetics, gene editing, or homologous recombination provide an auxiliary means to develop next generation gene-deleted vaccines, which is coming closer to the market in the short-term. 

In view of the recent five years of research on gene-deleted vaccines (Table 3), most of them can resist the attack of homologous parental strains after vaccination. In 2015, O’Donnel et al. vaccinated pigs with a low dose of the virulent strain Georgia 2007 with the 9GL gene-deleted, which can induce complete protection of pigs against homologous parental viral attack at 28 days post immunization. Simultaneously, the team knocked out six genes of MGF360/505, multigene families 360 and 505 of Georgia 2007, and obtained candidate vaccine strains with similar replication ability as parental virus but fully attenuated in pigs, which could completely resist the attack of parental strains after vaccination. Moreover, a deleted strain after knockout the 9GL and UK virulence genes could provide homologous protection only at 14 days post immunization [57,71,74]. Additionally, Reis et al. (2017) reported that, after knocking out the DP148R of Benin97/1, an attenuated strain with similar replication ability to the parent virus was obtained, which could induce high levels of protection against the homologous strain [77]. In a study by Gallardo et al. (2018), the recombinant NH/P68 strain lacking the A238L gene showed ideal protective effect against the homologous virulent strain L60, however, some vaccinated pigs had certain viremia and side effects [79]. Therefore, although the gene-deleted vaccines have achieved some success, there are still some uncertainties that deserve consideration. 

In our vision, the same gene deletion in different strains do not invariably have the identical outcomes, because the effect on the attenuation and protection against ASFV may be strain dependent. In 2020, Chen et al., found that unlike Georgia 2007, the Chinese HLJ/2018 virus (HLJ/18-9GL&UK-del) with a similar deletion of 9GL and UK was attenuated in pigs, but could not provide any protection against homologous virus challenge. The researchers have shown that the molecular basis of the virulence of ASFV may vary among strains, and that the biological changes caused by gene deletion in one ASFV strain may differ in other strains [41]. In 2017, Monteagudo et al. found that the virulence of BA71ΔCD2 obtained by artificially deleting the CD2v gene of the genotype I ASFV stain BA71 was significantly reduced, and this candidate strain was not only resistant to the homologous parental virulent strain (BA71), but also provided certain cross-protection to heterologous virulent strains (E75, Georgia 2007/1). Ultimately, the induced immune protection was dose-dependent and correlated with the ability of BA71ΔCD2 to induce specific CD8^+^ T cells in vitro [80]. Unexpectedly, deletion of the CD2-like gene from the genome of ASFV II Georgia/2010 does not attenuate virulence in swine [81]. This inconsistency in results may be attributed to a variety of factors, including discrepancy in animal genetics and viral strains, etc., and the diversity of ASFV isolates with different virulence complicates the assignment of ascertaining the determinants of immune protection. Furthermore, for the same strain, when selecting deleted genes, it is uncertain whether deleting more virulence genes simultaneously, and its protective effect, is more ideal. For example, in 2016, O’Donnell’ team further deleted MGF360/505 on the basis of ASFV-Georgia 2007/1-Δ9GL, although the deleted strain was highly attenuated, it had no protective effect on the infection of homologous virulent strains [56], and the same problem occurred when Ramirez-Medina et al. simultaneously deleting 9GL, UK, and NL genes of the Georgia/2007 strain [82]. Overall, the related mechanism is still much less explored, and researchers need to optimize deletable gene combinations in further work to fill the gap in the study of related virulence or immune escape genes.

Although most of the gene-deleted vaccines without ASFV-related virulence genes can provide complete protection, there is still a risk of virulence returning due to the residual virulence. The new investigation showed that the attenuated strain HLJ/18-6GD obtained by deletion of six genes of MGF360/505, multigene families of China HLJ/2018 strain, could protect pigs from the attack of parental virulent stains. However, its safety assessment data indicated that HLJ/18-6GD has a high risk of converting to a virulent strain. In the group’s latest study, the HLJ/-18-7GD, which further deleted the CD2v gene, was obtained to eliminate this risk, fully attenuated, and with a low risk of converting to a virulent strain, and could provide complete protection in pigs against lethal ASFV challenge. HLJ/-18-7GD is a safe and effective vaccine against ASFV [41] and, as the most promising vaccine candidate strain in China, is expected to play an important role in controlling ASFV transmission. The safety of ASF gene-deleted vaccines is critical for practical application, therefore, it is necessary to study the genetic stability and determine the safety and immunogenicity of the vaccines within a certain dose range. 

At present, the main factors restricting the development of ASFV gene-deleted vaccines are still insufficient research on virulence-related genes and immune escape mechanisms of ASFV, as well as subclinical symptoms and viremia caused by vaccination. A recent study described that a method of rapid isolation and purification of the deleted strain by combining the conventional homologous recombination with fluorescence-activated cells sorting (FACS) may greatly facilitate studies on understanding ASFV gene functions and accelerate the development process of recombinant live attenuated vaccine to some extent [41]. It is believed that the optimal combination of gene-deleted vaccines can be obtained on the basis of clarifying the biological functions of more unknown genes of ASFV.

### 5.3. Subunit and DNA Vaccines

At present, ASF genetically engineered vaccines, such as subunit vaccines, DNA vaccines, and virus vectored vaccines, provide a targeted approach with fewer side-effects and higher safety. As is shown in Table 4, We summarized ASF genetically engineered vaccines evaluated in recent years. Several ASFV proteins (p30, p54, p72, CD2v, EP153R, p12, D117L, pp62, etc.) have been studied and reported as the main targets of ASF vaccines, and the immune protective ability of these proteins has been tested, including individual ASFV antigen target or multi-target cocktail vaccination. 

It was previously thought that ASFV could not induce neutralizing antibodies. Gomez-Puertas et al., found that p72, p54, and p30 proteins have neutralizing effects, p72 and p54 could inhibit virus adsorption, p72 and p30 could activate cytotoxic T lymphocyte (CTL) response, while p30 could inhibit virus internalization [85]. Other envelope or endomembrane proteins of ASFV, such as CD2v, p12 and D117L, may also induce neutralizing antibodies and be involved in inhibiting viral invasion and release [86]. Ruiz-Gonzalvo et al., used baculovirus to express CD2v and vaccinated pigs with Freund’s adjuvant. The pigs had dose-dependent protective effects against the attack of the homologous ASFV E75 strain without neutralizing antibodies, revealing that factors other than neutralizing antibodies played an additional role in ASFV protection, which may be associated with CD2v-induced antibodies that could inhibit erythrocyte adsorption and temporarily inhibit infection [87]. Researchers used recombinant baculovirus-expressed ASFV p30 and p54 to vaccinate pigs, which could stimulate the body to produce neutralizing antibodies, and had a certain immunological protective effect against ASFV E75 strains [88]. Subsequently, the similar outcome occurred in pigs vaccinated with the chimeric protein p54/30 expressed by recombinant baculovirus [89]. However, pigs vaccinated with a mixture of p30, p54, p72, and p22 expressed by baculovirus could not resist the attack of the virulent ASFV Pr4, though ASFV-specific neutralizing antibodies could be detected in the pigs with delayed clinical symptoms and reduced viremia [90]. Neutralizing antibodies against these proteins are not sufficient to elicit antibody-mediated protection. Therefore, it brought on doubts about the relevance of p30, p54, and p72 in immune protection and debates about antibody-mediated neutralization protection. Recently, the ASFV serological determinants determined so far have recently been listed by an expert group, and a summary review has been published [91]. In 2011, Argilaguet et al. vaccinated pigs separately with pCMV-PQ, a plasmid encoding p30 and p54, two immunodominant structural viral antigens, and pCMV-APCH1PQ, a plasmid encoding p30, p54 fused with a single chain variable fragment of an antibody specific for a swine leukocyte antigen II (SLA-II). The experiments showed that both of the plasmids were not able to confer protection against lethal challenge with the virulent E75 ASFV-strain, and a viremia exacerbation was detected in each of the pigs correlating with the presence of non-neutralizing antibodies. The results clearly demonstrate that the adjuvant effect of SLA-II, which could enhance the immune response induced in pigs, and confirmed the critical role of CD8^+^ T cells in the response to ASFV [92]. In 2012, the group constructed the plasmid pCMV-sHAPQ, which fused the extracellular domain of ASFV Hemagglutinin/CD2v (sHA) with p54 and p30. After immunizing pigs with the expressed product, strong humoral and specific T cell responses in pigs was induced, which demonstrating the potential adjuvant properties of sHA, but no neutralizing antibody was detected and the pigs cannot resist the attack of virulent strains yet, which might be that the low levels of non-neutralizing and exacerbating antibodies in turn could counteract the protective effects of the specific CD8-T cells induced by the vaccine. Subsequently, the team designed a new recombinant plasmid pCMV-UbsHAPQ encoding three viral determinants (sHA, p54, and p30) of ASFV fused with ubiquitin to improve the presentation of MHC-I and enhance the induced CTL response while avoiding the induction of antibodies, and found that the recombinant plasmid could induce a strong CTL response in the absence of antibodies and protect 2/6 pigs from lethal challenge with ASFV E75 stains. Furthermore the protection may be associated with the proliferation of HA-specific CD8^+^ T cells [93]. Additionally, Lacasta et al. constructed a random DNA library containing 80 ASFV open reading frames (ORFs) fused with Ub, which indicating the presence of other determinants that induce protective CD8^+^ T cells and revealing the potential protective capacity of new ASFV antigens including CP312R [94]. The construction of expression libraries helps to thoroughly determine immunogenicity and virus-driven host immune regulatory factors.

At present, most of the protective antigens of ASFV that used in current subunit vaccines are insufficient to provide complete protection for vaccinated pigs. On the whole, with only a few ASFV proteins as antigens, it is difficult to provide effective immune protection even if neutralizing antibodies are emerged [95]. Although DNA vaccines can induce high levels of specific T cell responses in the host [91,93], they still cannot fully resist the challenge of virulent strains. The ASFV coding genes screened by DNA vaccination may play an established role in dissecting the immune mechanism and participating in the protection of ASFV antigens, but there is still a long way to go to achieve the goal of providing effective immune protection.

### 5.4. Virus Vectored Vaccines

A large number of studies have demonstrated that virus vectored vaccine can induce strong specific antibody-mediated humoral responses and IFN-γ secreting cellular responses in pigs, which can provide partial effective protection against ASFV challenge [96,97,98]. In 2019, as shown in Table 4, Lokhandwala’s group evaluated the immune response and protective effect induced by two recombinant adenovirus combinations, Ad-ASFV-I (A151R, B119L, B602L, EP402R∆PRR, B438L, K205R, A104R, pp62, and p72) and Ad-ASFV-II (p30, p54, pp62, p72, and pp220), by intranasal challenge of ASFV-Georgia 2007/1 on the basis of previous studies. Vaccination of pigs with Ad-ASFV-I in combination with BioMize0226 adjuvant revealed that although strong ASFV antigen-specific IgG responses were induced, vaccinated pigs were not able to resist the challenge of the virulent strain Georgia 2007/1 and developed an enhanced immune-response dependent disease. Thus, which factors play a role is unsolved, while high levels of antibodies seem to have some contribution. Additionally, two adjuvants (BioMize0226 and ZTS-01) were used to evaluate Ad-ASFV-II in this study, interestingly, in the presence of BioMize0226 adjuvant, ASFV-II induced much higher antigen-specific antibody response, but 8/10 vaccinated pigs died from virulent strain challenge. In contrast, the antibody response induced by ZTS-01 preparation was even weaker, paradoxically, 5/9 vaccinated pigs could resist the attack of homologous virulent strains, and survivors showed mild clinical symptoms and no viremia (non-statistical significance). The contradictory results in this study show that immune-induced antibody responses are not necessarily proportional to protective immunity, highlighting the complexity of the role of antibody responses in anti-ASFV, and also reflecting that different adjuvants may induce different innate immune responses, which require further study and evaluation of the efficacy of neoantigens formulated in appropriate adjuvants [99]. Moreover, Murgia et al. discovered that the strategy enhanced with natural attenuated strain OURT88/3 could broaden the recognition of ASFV epitopes, but its protective efficacy needs further verification [100]. In Hubner’s group, the rapid evolving genetic manipulation platform based on CRISPR/Cas9 provides a new method for screening potential immune protective antigen for more effective development of ASF virus-vectored vaccines [101]. 

Limited understanding of what constitutes a protective and pathogenic immune response to ASFV has hindered the development of virus vectored vaccines, immune hyperstimulation seems to be a key factor affecting the course of ASF, and high levels of antibodies seem to have a particularly adverse impact on clinical outcomes and protection, and whether the focus should be shifted to less immunogenic ASFV antigens remains to be further explored.

### 5.5. Combined Vaccination Strategy

Given the fact that neither subunit nor DNA vaccine vaccination can provide complete protection, researchers have proposed new vaccination strategies, including “cocktail” vaccination, and “Prime-Boost” vaccination strategies that can rapidly generate large numbers of memory CD8^+^ T cells, after understanding the key role of simultaneously inducing antibody-mediated immune responses and CD8^+^ T cells in immune protection.

In the previous discussion, we found that DNA vaccines can induce broad-spectrum immune responses, and DNA-protein immunity may help to identify virus antigen-specific responses, which can provide more potential information for the development of more efficient and safer ASFV subunit vaccines. In 2019, the combination of ASFV recombinant protein (p15, p35, p30, p54, CD2v-E) and pcDNA3.1 encoding ASFV gene (CD2v, p30, p72, CP312R) was selected to immunize pigs with ISA25 adjuvant. The results showed that pigs vaccinated with ASFV DNA+ protein could activate cell-mediated immune response, in which p72 was the main inducer of IFN-γ [102]. In the current study, the mechanism of synergy between specific ASFV proteins and pcDNAs encoding ASFV genes is elusive, and it is likely that pcDNAs trigger some nonspecific immune responses, thereby enhancing the specific response to these proteins. At the same period, Sunwoo et al. vaccinated pigs three times with a mixture of ASFV plasmid DNA (CD2v, p72, p32, +/−p17) and recombinant protein (p15, p35, p54, +/−p17). The vaccinated pigs, although producing antigen-specific antibodies, they lacked the ability to neutralize the virus, and as a result, were unable to resist ASFV Armenia 2007 strain challenge, and markedly enhanced pathological changes were observed in vaccinated pigs [103]. Immune-mediated increases in ASFV infection and disease have occurred in several vaccine challenge studies, including those mentioned above, and these ASFV immunogens are likely to cause more severe symptoms due to antibody-dependent enhancement (ADE) affecting neutralizing antibody production.

In recent years, vaccination of pigs with complex ASFV antigen preparations was carried out in order to find an appropriate balance between antibody and cell-mediated ASFV immune response. In 2017, Lopera-Madrid et al., found that vaccination with HEK 293-purified recombinant ASFV proteins (p72, p54, p12) could promote the production of ASFV-specific antibodies, but did not enhance cellular immunity. However, enhanced vaccination with Modified vaccinia virus Ankara (MVA)-vectored antigens (p72, EP153R and CD2v) could complement the former and promote the production of IFN-γ by cellular immunity [104]. On the basis of DNA vaccines, a strategy of DNA prime and recombinant vaccinia virus (VACV) boost has been proposed in order to orient the encoded antigens to specific antigen presentation pathways to enhance the induction of protective responses, and has also been widely used to screen the immunogenicity and potential protective antigens of ASFV. In 2018, Jancovich et al. identified a subset of ASFV antigens including p30, p72, and D117L that could effectively stimulate humoral and cellular immunity by screening 47 viral genes with this method. However, after the challenge of Georgia 2007/1, despite the viral load in blood and lymphoid tissues of vaccinated pigs decreased significantly, all vaccinated pigs developed acute ASF, and more severe clinical and pathological signs were observed [105]. On the basis of previous work, researchers proposed recombinant adenoviruses (r Ad) prime and MVA boost as improved delivery systems. In 2019, Netherton et al. screened 12 proteins that could induce ASFV-specific cellular immune response in pigs from ASFV OUR/T33 strain. Accordingly, five ASFV antigens, MGF110-4L, MGF110-5L, M448R, C129R, and I215L were defined as good antibody inducers by r Ad prime and enhanced vaccination with MVA virus encoding the same antigen, further targeting the determinants of ASFV cellular immune response. Although this candidate vaccine could not protect vaccinated pigs from acute ASFV OURT88/1 challenge, some vaccinated pigs showed delayed clinical symptoms and reduced viremia [49]. Similarly, in May 2020, Goatley’s group selected different antigen pools of viral vectors to induce the production of ASFV-specific antibodies and cellular immune responses. In this study an antigen pool consisting of eight ASFV adenoviral vectors, where the ASFV genes B602L, B646L/p72, CP204L/p30, E183L/p54, E199L, EP153R, F317L, and MGF505-5R were embedded, respectively, were used to immunize pigs, and boost with MVA virus encoding the same antigen. Two animal trials were carried out successively, with promising results [42], in the second animal experiment, 6/6 of pigs were protected from lethal doses of virulent ASFV I strain. In fact, the number of survivors after vaccination with the same antigen pool differed between the two trials, presumably because the immune dose of recombinant viral vectors was increased and an overreaction of the immune system were treated with flunixin meglumine (a non-steroidal anti-inflammatory and antipyretic drug) on day 5 after challenge, compared with experiment 1, which provided 2/6 protection. On the whole, this research achievement is an unprecedented breakthrough since the development of ASFV live-vectored vaccine.

What antigen can play a protective role and how to orient the antigen to a specific presentation pathway are the urgent problems to be solved in the development of ASFV genetically engineered vaccine at present. Combining the diversity of existing virus strains, further exploring the potential protective antigens of ASFV and the optimal immune mechanism to be triggered after vaccination, the use of multiple ASFV antigens or antigen fragments, adjuvants, and complex formulations of expression vectors are a new paradigm for the development of ASFV genetically engineered vaccines.

## 6. Discussion and Conclusions

Since the first occurrence of ASF in China in August 2018, the epidemic has spread continuously and rapidly throughout the country. The wide distribution of wild boars and soft ticks in China may make it easier to form a natural epidemic focus of ASFV. In addition, the diversified breeding patterns, high breeding density, and weak biosafety awareness of practitioners make the situation of prevention, control, and eradication of ASF in China very serious. As there is still no commercial vaccine available, the domestic strategy for ASF prevention and control relies largely on the strict biosecurity system. Developing safe and effective animal vaccines will help control and eradicate ASF from wild boars and domestic pigs in China. 

Based on the above analysis of the global ASF vaccine research status, the recombinant LAVs developed with the attenuated strain of ASFV virulence gene knockout has the most optimistic prospects as a short/medium-term vaccine candidate in the future. However, before practical application, comprehensive evaluation of the safety risks, including virulence re-enhancement, adverse reactions, and persistent infection of the candidate strains, is needed. In addition, it is necessary to develop a matched diagnosis technology of DIVA, the establishment of ASFV culture passage cell lines, and small animal infection models for vaccine research and production, and, most importantly, basic research on how viruses regulate host responses to infection and the role of virus-encoded proteins in evading host defenses will contribute to developing the next generation of recombinant LAVs. At the same time, ASF subunit vaccines, DNA vaccines, and live virus-vectored vaccines have great research potential with tremendous advantages in safety and DIVA diagnostic technology, but we must consider that although these vaccines can induce strong antigen-specific humoral and cellular immune responses, they can rarely provide complete immune protection against lethal ASFV strains. Undoubtedly, we need to identify more antigens with protective potential and, on this basis, explore the combinations of antigens to induce high levels of immune protection. Moreover, the delivery systems and vaccination strategies need further optimized. Specifically, we must fully understand the structure and function of the main proteins of ASFV, the mechanism of infection and immunity, the recognition of the main immunogens and vaccine targets in order to conduct a more in-depth study of genetically engineered vaccines, and comprehensively evaluate their safety and immune effects in targeted animals. Ultimately, the mechanism of natural hosts against ASFV, including wild boar and warthog, will be helpful to target key factors in the development of ASFV vaccine. In conclusion, there is still a long way to go in the development of the ASFV vaccine.

The introduction of ASFV has led to a heavy loss of pig production capacity and a devastating hit to the pig industry in China, and has disrupted the original breeding structure and layout of pig raising in China, which has a significant impact on the original pig breeding mode and business model. Nevertheless, it is also an opportunity of rebirth for the pig industry. On the one hand, it improves the threshold of breeding, promotes the transformation and upgrading of the pig industry, and accelerates its process of scale, automation, and intelligence. On the other hand, it changes the concept and enhances the ability of epidemic prevention and control, improves the level of biosecurity, rebuilds the epidemic prevention team, and remodels the epidemic prevention system and the modern pig industry.

At the present stage, China has initiated special studies on major basic scientific issues of ASFV, vaccine creation, detection technology, epidemiology, special disinfectants, and vector control, which provide important scientific and technological support for ASF prevention and control in China. In addition, the phased results of ASF vaccine research and development in China have greatly encouraged the confidence to overcome ASF. As stated in the report on the situation of ASF from the World Organization for Animal Health (OIE), interregional cooperation should be encouraged to combat the spread of this cross-border transmitted disease. Controlling ASF will be a challenging long-term battle that requires the joint participation and coordination of all national and international stakeholders.

## Figures and Tables

**Table 1:** The distribution of ASF outbreaks in China, August 2018–July 2020.

Region	Provinces	Outbreaks	No. of Susceptible	No. of Incidence	No. of Death	Total Outbreaks	Outbreaks-ASF, %
Southwest	Sichuan	14	1873	308	247	46	25.84% (46/178)
	Guizhou	10	1763	259	215		
	Yunnan	13	2861	1155	921		
	Tibet	3	/	/	55		
	Chongqing	6	770	24	78		
Northeast	Liaoning	21	425.63	2276	2087	30	16.85% (30/178)
	Jilin	4	1458	196	204		
	Heilongjiang	6	746.49	5044	4158		
Central	Hubei	9	2026	167	121	23	12.92% (23/178)
	Hunan	8	134.43	729	400		
	Henan	3	260	178	94		
	Jiangxi	3	463	75	63		
Northwest	Ningxia	4	465	43	29	22	12.36% (22/178)
	Xinjiang	3	1124	204	146		
	Qinghai	2	101	46	31		
	Shanxi	6	119.06	459	266		
	Gansu	7	111.61	732	612		
Eastern China	Shandong	1	4504	17	3	21	11.80% (21/178)
	Jiangsu	4	690.83	3087	1469		
	Anhui	9	110.18	586	358		
	Zhejiang	2	/	486	396		
	Fujian	3	222.47	147	123		
	Shanghai	1	314	50	11		
Northern China	Beijing	4	140.50	138	129	19	10.67% (19/178)
	Tianjin	2	1000	292	256		
	Hebei	1	5600	/	/		
	Shanxi	5	8379	178	100		
	Inner Mongolia	7	995	348	311		
Southern China	Guangdong	3	6167	1681	31	17	9.55% (17/178)
	Guangxi	8	278.39	129	966		
	Hainan	6	2162	432	223		

“/”, representing no relevant information.

**Table 2:** Advances in major virulence genes/immune escape-related genes of ASFV.

Pathway	Encoded Protein/Genes	Essential/Nonessential	Mechanism	Reference
Regulation of host protein expression	A238L	Nonessential	Inhibits NF-κ B and NFAT activation	[51]
	**NL** **(DP71L)**	Nonessential	Inhibition of activation of peIF2α-ATF4-CHOP signaling pathway and its mediated apoptosis	[52,53]
Interference with innate immune system	**MGF360,MGF505/530**	Nonessential	Inhibition of transcription of type I IFN and other cytokines Increased survival of infected cells	[54,55,56,57]
	I329L	Nonessential	Inhibits TLR signaling Inhibiting NF-κ B and IRF3 signaling pathways	[50,58,59]
Regulation of apoptosis/autophagy	A179L	Nonessential	A member of the Bcl-2 family Inhibition of apoptosis in the early stage of infection Inhibition of cell autophagy	[60,61]
	4CL (A224L)	Nonessential	Inhibition of TNF-alpha-induced caspase 3 activation and apoptosis	[54]
	EP153R	Nonessential	C-type lectin, participate in the process of blood cell adsorption Regulating apoptosis and inhibiting MHC-1 expression	[62,63,64]
	P54 (E183L)	Nonessential	Participate in viral particle assembly and viral adhesion to host cells Induce apoptosis in the late stage of infection	[65,66]
Others	**CD2v** **(EP402R)**	Nonessential	Mediate erythrocyte adsorption and promote virus transmission Interacts with cellular AP-1 protein and participates in intracellular transport of virus Inhibition of lymphocyte proliferation	[58,67,68,69]
	L83L	Nonessential	Binding host protein IL-1beta to inhibit its antiviral effect	[70]
	**UK** **(DP96R)**	Nonessential	Negative regulation of type I IFN expression and NF-κ B signaling by inhibition of TBK1 and IKKβ	[71,72]
	**9GL** **(B119L)**	Nonessential	Influencing virion maturation and viral growth in macrophages and viral virulence in swine	[56,71,73,74,75]
	**TK** **(A240L)**	Essential	Determines virulence	[76]
	**DP148R**	Nonessential	The function is elusive yet. Deletion of DP148R greatly reduces viral virulence	[77,78]

Bold markers are virulence-determining genes encoded by ASFV that have been reported so far.

**Table 3:** Overview of ASF gene-deleted vaccine research (2015–2020).

Source Strain	Genotype	Deletion Protein (Gene)	Cell	Virulence Changes	Protection	Reference
Benin 97/1	I	DP148R, CD2v (EP402R), EP153R	PBMs, WSL-R ^1^			[78] (2020)
OURT88/3 (attenuated)	I	I329L	PBMs		Reduced	[50] (2020)
Georgia/2007	II		PBMs	No attenuated	No	
China HLJ/18	II	MGF360/505 ^3^, CD2v (EP402R)	PBMs	Attenuated	Homologous	[41] (2020)
China HLJ/18	II	9GL, UK	PBMs	Attenuated	No	
China HLJ/18	II	MGF360/505 ^3^	PBMs	Attenuated	Homologous	
Georgia/2010	II	CD2v(EP402R)	Primary macrophages	No attenuated	No	[81] (2020)
Georgia/2007	II	9GL (B119L), UK (DP96R), NL (DP71L)	Primary macrophages	Attenuated	No	[82] (2019)
NH/P68 (attenuated)	I	A238L	COS-7 ^2^	Highly attenuated	Homologous (I L60) No heterologous (II Arm07)	[79] (2018)
Benin 97/1	I	MGF360/530/505	PAMs	Attenuated	Homologous	[55] (2016), [83] (2018)
Benin 97/1	I	DP148R	PAMs	Attenuated	Homologous	[77] (2017)
BA71	I	CD2v (EP402R)	COS-1 ^2^	Highly attenuated	Homologous and heterologous (I E75, II Georgia 2007/1)	[80] (2017)
Georgia/2007	II	9GL, UK	Primary macrophages	Fully attenuated	Homologous	[71] (2017)
ASFVG/VP30	II	TK	Primary macrophages, Vero	Attenuated	No	[76] (2016)
Pr4	II	9GL	Macrophages	Fully attenuated	Homologous	[84] (2016)
Georgia/2007	II	9GL, MGF360/505 ^3^	Primary macrophages	Highly attenuated	No	[56] (2016)
Georgia/2007	II	MGF360/505 ^3^	Primary macrophages	Fully attenuated	Homologous	[57] (2015)
Georgia/2007	II	9GL	Primary macrophages	Attenuated	Homologous	[74] (2015)

^1^ WSL-R: Spontaneously immortalized wild boar cell line; ^2^ COS-7 /COS-1: a monkey cell line transformed with the large antigen of SV40; ^3^ MGF360/505: Including MGF505-1R, MGF360-12L, MGF360-13L, MGF360-14L, MGF505-2R, MGF505-3R.

**Table 4:** Evolving strategies for ASFV genetically engineered vaccines.

Sequence Source	Gene/Protein	Vector/System	Adjuvant	Specific Antibodies	Neutralizing Antibody	Cellular Immunity	Protection	Reference
Protein-based subunit vaccines								
E75CV	HA (CD2v)	Baculovirus	Freund’s	Yes	No		Homologous protection (3/3) Dose dependent	[87]
E75	p54,p30	Baculovirus	Freund’s	Yes	Yes		Partial protection (3/6),	[88]
E75	p54/p30 chimera	Baculovirus	Freund’s	Yes	Yes		Homologous protection (2/2) Mild clinical symptoms	[89]
Pr4	p54,p30,p72,p22	Baculovirus	Freund’s	Yes	Yes		No (0/6) Delayed clinical disease Reduced viremia	[90]
E70	Group1:p158,p327,p14,p220 Group3:p30,p72	Synthetic peptides	Freund’s	No			No; Group1&3 Increased average survival Reduced mean viral titers	[95]
DNA vaccines								
E75	p54/p30 fusion	pCMV		No		No	No (0/4)	[92]
	p54/p30/SLA-II fusion	pCMV		Yes	No	T cell response	No (0/4) Viremia enhancement	
E75	sHA/p54/p30 fusion	pCMV		Yes (p54;p30)	No	IFN-γ	No (0/6);	[93]
	sHA/p54/p30/Ub fusion	pCMV			No	Strong CTL IFN-γ	Partial protection (2/6) The absence of viremia	
E75	80 ORFs fragments/Ub fusion	DNA expression library		Yes		Yes	Partial protection (6/10) Reduced virus titers	[94]
Virus-vectored vaccines								
E75	sHA ^1^/p54/p30fusion	BacMam		No	No	IFN-γ	Partial protection (4/6) The absence of viremia	[97]
Georgia 2007/1	p30,p54,pp62,p72	Adenovirus	BioMize	Strong		IFN-γand CTL		[98]
Georgia 2007/1	A151R,B119L,B602L,EP402R∆PRR,B438L,K205R,A104R	Adenovirus	BioMize; ZTS-01	Strong		IFN-γ		[96]
Georgia 2007/1	Ad-ASFV-I: A151R,B119L,B602L,EP402R∆PRR,B438L,K205R,A104R,pp62,p72	Adenovirus	BioMize	Strong		IFN-γ	No Immune-response dependent enhancement of disease	[96]
	Ad-ASFV-II: p30,p54,pp62,p72,pp220 (p37-34-14,p150-I,p150-II)		BioMize	Higher		IFN-γ	Partial protection: (2/10)	
			ZTS-01	Lower		IFN-γ	Partial protection: (5/9) Lower clinical score The absence of viremia	
Combined vaccination strategy								
Georgia 2007/1	p72, p54, p12	HEK 293cell	TS6	Yes	No	Less T Cell response		[104]
	p72, C-type Lectin (EP153R), CD2v	MVA ^2^	TS6	No		T Cell response		
	p72, C-type Lectin (EP153R), CD2v	r VACV ^3^ prime + protein boost	TS6			T Cell response IFN-γ		
Georgia 2007/1	47 antigens	DNA prime + r VACV boost	CpG oligo	Yes	No	T Cell response	No Reduced viral load Higher clinical scores	[105]
E70;Ba71V	DNA:CD2v,p30,p72,CP312R; Proteins: p15, p35, p54, p72, CD2v-E (s HA)	DNA+ Protein	ISA25	Yes	20%; 10%	Some		[102]
Georgia 2007/1; Ba71V	DNA:CD2v, p72, p30, +/-p17; Proteins: p15, p35, p54, +/-p17	DNA+ protein	ISA25	Yes	No	Some	Challenge: Armenia 2007 No Disease enhancement	[103]
Ba71V	p30, p54, p72, s HA/p72	Alphavirus RPs ^4^		Yes				[100]
	p30 (Ba71V) + OURT88/3	Alphavirus RP prime + LAV boost		Yes	Yes			
OUR T88/3	A151R, p72, C129R, p30, p54, E146L, I215L, I73R, L8L, M448R, MGF110-4 L, MGF110-5 L	r Ad prime + MVA boost		Yes		Yes	Challenge: OUR T88/1 No Reduced and delayed clinical signs; Reduced viremia and viral load	[49]
OUR T88/3 Benin 97/1	p72, p30, p54, E183L, E199L, EP153R, F317L, MGF505-5R	r Ad prime + MVA boost	No	Yes (Expect:B646L,E183L,EP153R)	Some (E183L,CP204L)	IFN-γ	Challenge: OUR T88/1 Exp.2 (6/6) Reduced Viremia Infectious virus persisted Exp.1 (2/6)	[42]

^1^ sHA = Extracellular domain of ASFV hemagglutinin; ^2^ MVA = Modified vaccinia virus Ankara; ^3^ VACV = Vaccinia virus; ^4^ RPs = Replicon particles.

## Abbreviations

ASF	African swine fever
ASFV	African swine fever virus
OIE	The World Organization for Animal Health
MGF	Multigene families
MARA	Ministry of Agriculture and Rural Affairs of the People’s Republic of China
CSF	Classical Swine Fever
HRP	High-risk periods
TADs	Transboundary animal diseases
DIVA	Differentiating infected from vaccinated animals
LAVs	Live attenuated vaccines
FACS	Fluorescence-activated cells sorting
PBMs	Peripheral blood monocytes
WSL-R	Spontaneously immortalized wild boar cell line
COS-7/COS-1	A monkey cell line transformed with the large antigen of SV40
PAMs	Pulmonary alveolar macrophage
CTL	Cytotoxic T lymphocyte
SLA-II	Swine leukocyte antigen II
ORFs	Open reading frames
ADE	Antibody-dependent enhancement
MVA	Modified vaccinia virus Ankara
VACV	Vaccinia virus
r Ad	Recombinant adenoviruses
